# Vesicle Fusion as a Target Process for the Action of Sphingosine and Its Derived Drugs

**DOI:** 10.3390/ijms23031086

**Published:** 2022-01-19

**Authors:** José Villanueva, Yolanda Gimenez-Molina, Bazbek Davletov, Luis M. Gutiérrez

**Affiliations:** 1 Instituto de Neurociencias, CSIC-Universidad Miguel Hernández, Cra de Valencia S/N, Sant Joan d’Alacant, 03550 Alicante, Spain; ygimenez@umh.es; 2 Department of Biomedical Science, University of Sheffield, Sheffield S10 2TN, UK; b.davletov@sheffield.ac.uk

**Keywords:** sphingosine, FTY-720, exocytosis, vesicle fusion, mitochondria, neurotransmitter release, neuroendocrine cells

## Abstract

The fusion of membranes is a central part of the physiological processes involving the intracellular transport and maturation of vesicles and the final release of their contents, such as neurotransmitters and hormones, by exocytosis. Traditionally, in this process, proteins, such SNAREs have been considered the essential components of the fusion molecular machinery, while lipids have been seen as merely structural elements. Nevertheless, sphingosine, an intracellular signalling lipid, greatly increases the release of neurotransmitters in neuronal and neuroendocrine cells, affecting the exocytotic fusion mode through the direct interaction with SNAREs. Moreover, recent studies suggest that FTY-720 (Fingolimod), a sphingosine structural analogue used in the treatment of multiple sclerosis, simulates sphingosine in the promotion of exocytosis. Furthermore, this drug also induces the intracellular fusion of organelles such as dense vesicles and mitochondria causing cell death in neuroendocrine cells. Therefore, the effect of sphingosine and synthetic derivatives on the heterologous and homologous fusion of organelles can be considered as a new mechanism of action of sphingolipids influencing important physiological processes, which could underlie therapeutic uses of sphingosine derived lipids in the treatment of neurodegenerative disorders and cancers of neuronal origin such neuroblastoma.

## 1. Lipids and Exocytosis

The fusion of vesicles with other lipid bilayers is essential for intracellular trafficking and release of neurotransmitters and hormones [1,2,3,4]. Release of neurotransmitters and hormones involves the active transport of the vesicles using cytoskeletal elements such as F-actin and microtubules [5,6], tethering and docking of the vesicles with the target membrane [7,8], and finally calcium-induced fusion of the membranes, resulting in the release of vesicular contents to the extracellular media via exocytosis [9,10].

Traditionally lipids have been regarded as structural elements playing a relatively minor role in the molecular mechanisms of exocytosis whereas proteins such as SNAREs (soluble N-ethylmaleimide sensitive factor attachment protein receptors) are thought to be the central elements that generate the specificity and force needed for overcoming the repulsion of the negative charges within lipid bilayers that oppose fusion [11,12,13,14]. Even so, membranes need to adopt curved shapes during fusion, which is heavily influenced by the molecular structure of lipid and lysophospholipids in particular facilitating formation of conic shapes that are amenable to fusion [15,16].

In addition, some lipid components aggregate to form microdomains that facilitate recruitment of the proteins that catalyse exocytosis. For example, phosphatidyl inositol 4,5-biphosphate (PIP2) microdomains seem to act as beacons for coordinating F-actin bundles involved in recruiting SNARE proteins, and are needed for the translocation of secretory vesicles to their specific docking sites [17,18,19,20]. Further, cholesterol accumulation into lipid rafts can contribute by organizing clusters of secretory proteins such as syntaxin-1 [21].

Furthermore, lipids can be incorporated into secretory proteins such as SNAP-25 via post-translational modification consisting in the acylation of cysteine residues by palmitate, a saturated 16-carbon fatty acid and in that way affect the location and the function of this protein [22]. It seems that the palmitoylation of 4 residues of this SNARE, increase the clustering of SNAP-25 in cholesterol and sphingomyelin rich lipid rafts, that could enhance the formation of secretory active sites thereby acting as a cohesive factor [23,24]. Moreover, some studies suggest that this modification exerts changes that enhance the forces acting on the zippering of the SNARE complex thereby enhancing the fusion of membranes [25], while others suggest that palmitoylation contributes simply to the anchoring role associated with the insertion of fatty acids in the membranes, as it has been traditionally accepted [26].

In addition to SNAP-25, other proteins participating in secretion such as synaptobrevin-2 have been demonstrated to be palmitoylated. This protein is modified by palmitoylation during development since the modification is only found in adult rats and not in embryonic cells [27]. Furthermore, one of the calcium sensors associated with membrane fusion, the protein synaptotagmin 1, incorporates fatty acid acylations in 5 residues close to the membrane anchoring domain [28]. Finally, the cysteine string protein (CSP), a molecular chaperone involved in protein folding [29], is heavily palmitoylated with 14 cysteine residues. These lipid residues have been found to be important for exocytosis in neuronal and endocrine cells [30,31].

As can be deduced from the multiple roles played by lipids mentioned above, their function in exocytosis is far more complex than that deduced from being the basic structural elements forming membranes, and this is further supported by recent data on the direct modulation of the secretory machinery by signalling lipids.

## 2. The Direct Interaction between Sphingolipids and SNAREs

As mentioned above there are several roles that could be assumed by lipids in order to influence the secretory process ranging from structural determinants to specific “markers” locating the position of exocytotic active sites. Nevertheless more recently it has been highlighted that certain type of lipids acting as intracellular signals, the so called signalling lipids, could interact directly with the SNARE proteins constituting the secretory machinery. Signalling lipids such as sphingosine or arachidonic acid (AA) are released from structural phospholipids by the action of phospholipases [32,33], and upon generation of saturated or polyunsaturated fatty acids (PUFAs), normally present in the sn-2 position, they diffuse freely to interact with either synaptobrevin in the case of sphingosine [34] or target syntaxin-1 in the case of AA [35].

These signalling lipids interact with a variety of parts of the secretory machinery such as SNAREs and in that way they modulate exocytosis. In this sense AA interaction with the t-SNARE syntaxin 1 was the first reported evidence of a direct interaction of signalling lipids with SNAREs linked to the exocytotic fusion machinery in 2005 [35]. In this study, the direct application of AA, or alternatively the treatment with PLA2s, was able to promote the formation of SNARE complexes in in vitro experiments using membrane preparations. Furthermore, this interaction occurs even in the “closed” conformation of syntaxin-1 promoted by the presence of Munc-18, suggesting that AA could access the hydrophobic core of syntaxin-1 when this protein forms part of the stable plasma membrane dimers with Munc-18 [35,36]. This AA interaction seems to be an essential aspect of syntaxins since it has been reported to occur in different syntaxins such as the 1 and 3 forms [36].

The AA regulation of the secretory machinery was further emphasized when it was reported that the protein α-synuclein accumulating in the neuronal cytoplasm during the pathogenesis of Parkinson disease, was able to sequester AA and in this way impede the activation of exocytosis caused by this signalling lipid [37]. Therefore, the physiological regulation of syntaxins by AA appears to be essential to sustain the correct levels of exocytosis and might be altered in neuronal disorders. 

**Figure 1:**
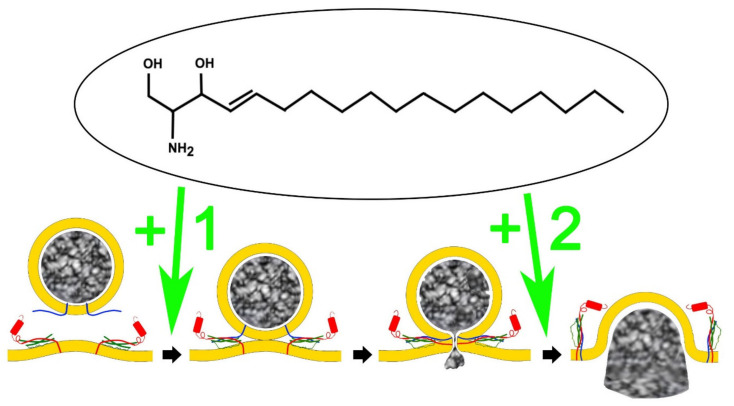
Sphingosine influences different steps of exocytosis. After the production of the signalling lipid sphingosine from sphingolipids, this modulates at least two phases of the exocytotic process, increasing the formation of the SNARE complex which enhance the frequency of vesicle fusions (1), and the transition from the “kiss and run” mode of fusion to the full collapse mode (2), causing an increase in the number of neurotransmitters released per event. Show here also the FTY720 structure.

Recently, by screening the ability of a variety of lipids in changing the formation of the SNARE complex in vitro it was proven that specifically sphingosine and some related lipids were able to induce synaptobrevin-2 binding to syntaxin-1 and SNAP-25 dimers formed in the target membrane [34] (Figure 1). Interestingly, L-sphingosine was as efficient as D-sphingosine in promoting SNARE complex formation indicating that this compound may alter the local membrane environment influencing synaptobrevin conformation. From the analysis of the sphingosine-related compounds assayed it was concluded that the length of the carbon chain and the presence of a positive charge in the head of the lipids were two essential features needed to promote SNARE-complex formation and the associated enhancement of the exocytosis [34]. The involvement of the vesicular SNARE synaptobrevin in the sphingosine effect was further emphasized when in synaptobrevin-2 knockout mice there was no evidence of secretory modulation by sphingosine [34]. Treatment of control cultured cells of neuronal and neuroendocrine origin with increasing concentrations of sphingosine resulted in dose-dependent enhancement of exocytosis with EC50 values around 10 µM [34]. A further step was taken to study the sphingosine modulation of SNAREs when the endogenous production of sphingosine was tested by using treatment with external sphingomyelinases (SMases) of nerve terminals [34] and cultured neuroendocrine chromaffin cells [38,39]. The results support the sphingosine role of synaptrobrevin-2 since treatment of these cell preparations with Botulinum Neurotoxin-D, which cleaves synaptobrevin-2 [34], prevented the release of neurotransmitters induced by SMases treatment.

Another study suggested that sphingosine could additionally act through a different molecular mechanism involving the SNARE syntaxin-1 [40]. In this scenario, sphingosine facilitates the formation of the dimer Munc-18-syntaxin-1, which may reduce the number of the vesicles in a competent state for fast fusion, thus somewhat opposing the conclusions obtained in the articles mentioned above. 

Interestingly, a physiological derivative of sphingosine, sphingosine-1P also has been found to alter the secretory response in neuroendocrine chromaffin cells [15,41] by a completely different mechanism; i.e., via modulating the amount of intracellular calcium. In this case, and in a recent report [42], this modified sphingosine appears to have a complex effect on exocytosis at different concentrations. At 1 μmol/L, sphingosine-1P, the Ca2+-activated K+ currents (IK(Ca) were inhibited in electrophysiology experiments whereas at a higher dose of 10 μmol/L the current was activated causing a remarkable increase in intracellular calcium and enhanced exocytosis [42].

Therefore, it is clear that the direct interaction of sphingosine or physiological derivatives such as sphingosine-1P with different elements of the secretory machinery may cause a complex modulation of the exocytotic response in neuronal and neuroendocrine cellular models, and that the complexity of such interactions deserves further study.

## 3. Sphingolipids Alter the Single Fusion Properties of Neurotransmitter Release

**Figure 2:**
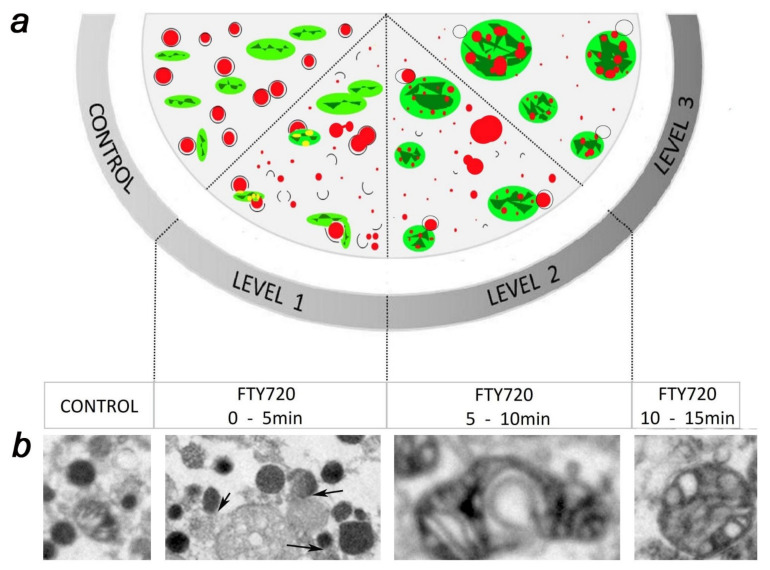
FTY-720 induces drastic changes in vesicles and mitochondria in chromaffin cells. (**a**) Schematic of the changes induced by 20 μmol/L FTY-720 incubation of cultured bovine chromaffin cells after a variety of incubation times, where the dense vesicles are depicted in red and mitochondria in green. (**b**) Micrographs showing examples of the changes by using electron microscopy. Three levels of changes are proposed: (1) Rapid formation of microvesicles from dense organelles (dark round organelles) and initiation of the fusion with mitochondria as indicated by arrows, (2) Heterotypic fusion of vesicles with mitochondria to form elongated mixed organelles, and (3) Formation of round mixed macroorganelles consisting of mitochondria incorporating the dense cores of several granules.

To study the way sphingolipids affect the secretory process, it is important to appreciate that this is a multi-step process starting with the translocation of the vesicles to the plasma membrane using active transport involving cytoskeletal elements [43,44], maturation of the docked vesicles to be competent for membrane fusion [8,45], and finally the fusion process itself that includes the opening of a fusion pore, subsequent dilation, and then the release of active substances that ends in full collapse of the vesicle into the plasma membrane [46,47,48] (Figure 2). Therefore, the use of biophysical techniques such as membrane capacitance methods [49,50], and amperometry [51,52], which resolve distinct stages of exocytosis, is essential for better understanding how sphingolipids alter the secretory pathway.

Our initial study describing the enhanced formation of SNARE complexes by sphingosine (Figure 1), also included the first biophysical observations of increased secretory responses in rat hippocampal neurons, melanotrophs, and chromaffin cells [34]. In this study published in 2009 the analysis of excitatory postsynaptic currents (EPSCs) in neurons was essential not only to show that sphingosine alters the fusion properties, but also that this effect was mediated by synaptobrevin-2.

Later on, two of the groups participating in this study and using distinct cellular systems studied more deeply the fusion exocytotic steps affected by sphingosine. First, and by using capacitance techniques to distinguish unitary fusion events in pituitary lactrophophs, Robert Zorec’s group demonstrated that sphingosine raises the frequency of fusion of both small vesicles and large dense granules [53]. In addition, they demonstrated that sphingosine promotes the full fusion mode of the larger vesicles while the smaller ones fused via the “Kiss and run” mode, thus only partially releasing their content. Therefore, it appears that sphingosine modulation depends on the size of the vesicles, and favors a distinct mode of fusion as shown in Figure 1.

We reached a similar conclusion using amperometry to measure catecholamines from cultured bovine chromaffin cells [38]. We used Sphingomyelinase to produce sphingosine and derivatives, which enhanced the number of events released in response to cell depolarization with a high concentration potassium solution. Moreover, the amperometry technique resolved the amount of neurotransmitter released per event and the kinetics of individual vesicle fusion, and additionally demonstrated that enzymatic sphingosine production enhances both parameters, which indicates that sphingosine promotes the full fusion of dense granules and that in control conditions, without added sphingosine, the release of catecholamines is only partial, indicating that it must be happening via the “Kiss and run” mode. Two years later, in collaboration with Dr. Alvarez de Toledo at the University of Seville we performed experiments using the whole cell and on-cell capacitance techniques to resolve the size of the vesicles fused and we found that sphingomyelinase treatment of rat chromaffin cells resulted in a clear increase in the frequency of fusions of both small and large vesicles without affecting the size of the vesicles measured by electron microscopy [39], in agreement with the results obtained in lactotrophops [53].

In a recent report, intracellular sphingosine-1P was also found to accelerate the rate of fusion pore expansion in chromaffin cells from mouse [54], thus indicating that not only sphingosine but also its derivatives may control directly the properties of membrane fusion. In agreement with this, the exocytosis from preparations of cortical granules from oocytes, has been found to be sustained by sphingosine and sphingosine-1P, thus the presence of a critical amounts of these sphingolipids appear essential to maintain normal levels of exocytosis [55].

It is important to note also that both sphingosine and sphingosine-1P might regulate other steps of the secretory process such us the initial entrance of calcium after cell stimulation [15,41], synapsin levels [56], and even the rate of membrane retrieval by endocytosis [57]. Therefore the possible stages of the exocytosis-endocytosis cycle regulated by sphingolipids is a subject of open discussion at the present.

## 4. An Analog of Sphingosine, FTY-720 (Fingolimod) Mimics Signalling Lipid Potentiation of Exocytosis

Not only sphingosine and its natural derivatives have been found to alter exocytosis in a variety of cellular systems as mentioned above, but more recently a fungi-derived sphingosine analog, FTY-720, also known as Fingolimod, has been reported to influence the secretory pathway. Initially, FTY-720 was employed as an immunosuppressant agent, since its phosphorylated form binds to the sphingosine-1P receptors [58], and thereby cause lymphocyte egress from the blood vessels and consequently immunosuppression [59]. Therefore, this drug was approved for the oral treatment of multiple sclerosis [60,61], the most common inflammatory disorder of the CNS [62].

Crucially, FTY-720, has been shown to induce the formation of the SNARE ternary complex in vitro, and, as a consequence, enhanced the exocytosis in neuroendocrine cells [63], thus mimicking the activity of sphingosine activity in neurosecretion. This drug at micromolar concentrations, as it happens with sphingosine, increases the frequency of glutamate release from rat synaptosomes, promotes the release of dense vesicles in melanotrophs and chromaffin cells, and augments neurotransmission from rat hippocampal neurons in culture. Interestingly, FTY-720 disrupts the interaction of the cytosolic part of synaptobrevin-2 with phospholipid membranes in in vitro assays, thus indicating that this SNARE mediates the molecular mechanism involving the FTY-720 effect, in agreement with the mechanism reported for sphingosine [34].

In a recent paper, we reported that FTY-720 has a complex effect on secretion from neuroendocrine chromaffin cells because, in addition to modifying the amount of quantal release and the frequency of vesicle fusion in cells stimulated by depolarizations, we also observed that the recruitment of vesicles in response to multiple pulses decreases, indicating that late phases of vesicle transport are also altered [64]. The result agrees well with a report in rat astrocytes in culture of a FTY-720 effect on the transport of the vesicles in the interior of the cytosol, limiting the mobility of different types of vesicles, and therefore their access to secretory sites [65].

In conclusion, it is possible that both sphingosine and FTY-720, in addition to altering the vesicle fusion properties in neuroendocrine and neuronal systems through the alteration of SNARE function, these small lipids could additionally alter the transport of vesicles through F-actin cytoskeleton, impairing vesicle access to secretory sites and that this could be related with the alteration of calcium dynamics by sphingolipids [55].

## 5. FTY-720 Induces the Heterotypic Fusion of Organelles

Our more recent study revealed that FTY-720 not only mimics sphingosine properties in relation to the fusion of vesicles and the plasma membrane during exocytosis but also exhibits new properties in relation to the fusion and fission of other cellular structures such as mitochondria that had not been reported previously [64].

Using confocal fluorescence microscopy of chromaffin cells, we detected substantial alterations in the shape and size of mitochondria that were accompanied by simultaneous alterations in the number and size of chromaffin granules. Fluorescent microscopy revealed that soon after adding 20 μmol/L of FTY-720 the fluorescently labelled vesicles decreased in size and, within 5 min of drug incubation, started to merge with mitochondria that had been labelled with a different dye. These merging organelles then increased in size and roundness at 15 min of initiation of the FTY-720 incubation. The changes were confirmed by electron microscopy that additionally showed a dramatic change in the fusion properties of chromaffin granules in the interior of the cellular cytoplasm [64] as seen in Figure 2. The sequence of events indicated that during FTY-720 treatment (Figure 2): (i) chromaffin granules in the cytosol initiate processes of homotypic fusion between themselves, and fissions to form microvesicles, (ii) with a small delay of minutes chromaffin vesicles started to fuse even with mitochondria increasing the size and roundness of these organelles, (iii) these heterotypic fusions ended in the formation of giant mitochondrial round structures containing multiple vesicular dense cores in their interior. To our knowledge, this type of mixed organelle product of the fusion of chromaffin granules with mitochondria had not been reported previously. Finally, the dramatic changes in mitochondria lead to a decrease of fluorescence of Mitotracker dye intensity and density revealing the impairment of mitochondrial redox function of around 70% at 15 min. of incubation with the drug [64]. This heavy loss of mitochondrial redox potential has a dramatic effect over chromaffin viability as it would be expected, causing 50% of the cells to die within 2 h after incubation with FTY-720 [64]. The cells do not recover, and 24 h after the treatment viability is reduced to 20% of the control non-treated cells [64].

Interestingly, the fusogenic alterations induced by FTY-720 were mediated by changes in the function of SNAREs since the expression of non-fusogenic SNAP-25 forms prevented the homotypic and heterotypic fusions [64]. Therefore SNAREs appear to participate in both, the “normal” exocytotic fusion, and the pathogenic homotypic and heterotypic fusions of organelles induced by FTY-720.

In summary, the results obtained with FTY-720 highlight that sphingolipid derived drugs might be acting on multiple targets involved in a variety of cellular and pathological processes, and that we need to consider that molecular mechanisms based in the alteration of SNARE fusogenic activity associated to exocytosis, homotypic, and heterotypic organelle fusion and fission processes could underlie the pathological alterations observed in neurons and neuroendocrine cells after application of sphingosine and derivatives.

## 6. The Potential Use of FTY-720 in the Treatment of Neuron-Related Syndromes and Cancers

In view of the important role of sphingolipids in neuronal and neuroendocrine signalling pathways [66], and the variety of cellular targets altered by FTY-720, as mentioned above, it is relevant to mention the possible use of this drug in the treatment of a variety of neuronal syndromes. This synthetic analogue of sphingosine has been extensively investigated for clinical use in the last decade, first, because of its anti-inflammatory properties, which, in its phosphorylated form, interacts with sphingosine-1P receptors [58], and affects many neuronal processes including neuroinflammation. Therefore, this drug was the first to be approved for the oral treatment of multiple sclerosis, which is the most common inflammatory disorder of the CNS [60].

In addition, a plethora of recent studies in a variety of animal models suggest that FTY-720 could have additional therapeutic applications ranging from the modulation of astrocyte and neuronal function, neuronal excitability, and even anti-cancer properties. Among the benefits, it has been shown that FTY-720 can stimulate neuronal gene expression, axonal growth and regeneration [67], and this is related to the improvement of neuronal function in multiple neuronal syndromes [68]. Specifically, FTY-720 has been found to attenuate excitotoxicity and neuroinflammation [69], has neuroprotective properties during ischemic episodes in murine models [70], and improves hippocampal synaptic plasticity and memory deficits [71]. It also attenuates social deficits, learning and memory impairments, and neuronal loss in a rat model of autism [72].

Regarding neurodegenerative disorders: It has been shown that FTY-720 enhances hippocampal synaptic plasticity and memory in Huntington’s disease models [73], attenuates beta-amyloid peptide-induced impairment of spatial learning and memory in rats [74], reduces dopaminergic degeneration in Parkinsonian models [75], and reduces synucleinopathy and neuroinflammation, restoring mitochondria function and behaviour [76].

The effects of FTY-720 on mitochondria—dramatically altering the membrane potential with the consequence of generating a rapid apoptosis that induces cell death—might be associated with effects on the proliferation of cells from cancers of neuronal origin that have been reported. For instance, FTY-720 induced apoptosis in neuroblastoma cells [77] and glioblastoma cells [78], with the reduction in the viability of the cells from medullablastome in a variety of murine models [79].

The anti-tumour effects of FTY-720 are observed in cancers from other, non-neuronal origins, for example in the treatment of leukaemia [80], with the effect being unrelated to sphingosine-1P receptors, but also in halting proliferation of breast cancer [81], melanoma [82], and myeloma cells [83], in a variety of mouse models.

**Figure 3:**
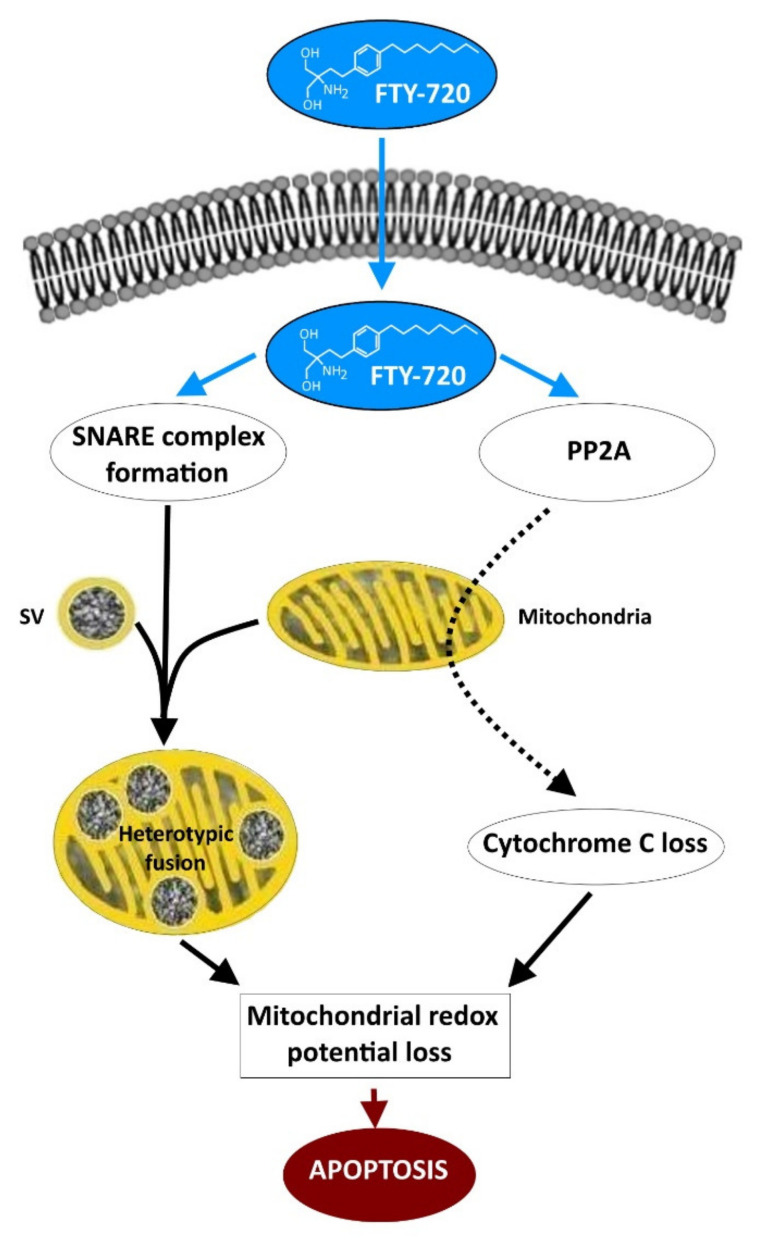
FTY-720 induces mitochondrial apoptosis by two different mechanisms. FTY-720 crosses the plasma membrane of neuronal and neuroendocrine cells to directly activate PP2A. Activation affects mitochondrial function by inducing the loss of cytochrome C, which is associated with the reduction of the mitochondrial redox potential. These major changes in mitochondrial activity precede the activation of apoptosis resulting in massive cancer cell death. In addition, FTY-720 enhances the formation of fusion complexes that induce the heterotypic fusion of dense synaptic vesicles (SV) with mitochondria. Again, this major change in mitochondrial structure and function results in the loss of the redox potential and the induction of apoptosis.

What could be the molecular mechanisms involved in the FTY-720 effect preventing cancer cell proliferation and inducing cellular death? Some studies indicate that this compound mainly induces mitochondria-involved apoptosis (see Figure 3) in a variety of types of cancer cells, since Bcl-2 overexpression prevents the FTY-720-induction of apoptotic stimuli [84]. Bcl-2 additionally controls the loss of the mitochondrial release of cytochrome c associated with a reduction in the mitochondrial membrane potential and causing the activation of caspases forming part of the apoptotic mechanism [85] as shown in Figure 3. In addition, FTY-720 induces G0/G1 cycle arrest in lymphoma lines that appears to be dependent of the activity of Protein Phosphatase type 2A (PP2A) [86], and in this study it was proposed that the molecular mechanisms governing the cell cycle arrest and the apoptosis mediated by FTY-720 are independent. It is tempting to think that either the apoptotic cascade induced by FTY-720, or the direct collapse of the membrane potential as a product of heterotypic fusion of mitochondria with granules, induced by this drug, could be used to trigger death of proliferating cells during cancer development (Figure 3).

Taken together, it is clear that FTY-720, an example of a drug that mimics sphingosine structure, could be used in the therapy of neuronal and neuroendocrine syndromes, and as an anti-tumour agent, due to the action upon a variety of molecular targets that influence the physiology of multiple cellular systems.

## 7. Conclusions

The role of lipids in exocytosis in neurons and neuroendocrine cells has been evolving dramatically in the last years to pass from being considered as merely structural components to a more active role in the direct regulation of the secretory molecular machinery [87,88,89].

In particular sphingosine, released from the structural membrane sphingolipids by the action of sphingomyelinases, and considered a signalling lipid, interacts directly with synaptobrevin-2, which promotes the formation of the SNARE complex, and increases membrane fusion by exocytosis in neuronal and neuroendocrine cells [34,38]. Furthermore, sphingosine seems to affect several steps in the secretory pathway to enhance the frequency of vesicular fusions [34] in a variety of cellular systems, and also to affect the single fusion kinetics to promote the full fusion mode of exocytosis in systems where there is a large proportion of partial neurotransmitter release by the “kiss and run” mode [39,53]. Other derivatives such as sphingosine-1P have also been implicated in the regulation of the fusion pore properties and are likely to be necessary to sustain physiological levels of neurosecretion [54,55].

The direct regulation of the secretory machinery by sphingosine and sphingosine-1P was further emphasized when a structural analog FTY-720, approved for the oral treatments of certain forms of relapsed multiple sclerosis [61], was shown to mimic the effect of sphingosine in enhancing neurosecretion [63] by affecting other stages of exocytosis. Interestingly, at higher micromolar concentrations, this compound also exhibited fusogenic properties in relation with intracellular organelles, promoting the homotypic fusion and fission of chromaffin granules in the interior of the cytosol, and the heterotypic fusion of these granules with mitochondria, causing major alterations in mitochondrial shape and size, a dramatic decrease in membrane potential, and finally, massive cell death [64].

Whether or not these effects on exocytosis are linked to the plethora of possible therapeutic uses of FTY-720 in the normal and pathological states is a matter that might be worth further study. This drug influences the activity of neurons and astrocytes by stimulating neuronal gene expression, axonal growth, plasticity, and regeneration [67]. In small doses it additionally reduces excitotoxicity and neuroinflammation [69], and has neuroprotective effects against ischemia [70]. Furthermore, FTY-720 improves neuronal deficits associated with major neurodegenerative disorders such as Huntington’s Disease [73], Parkinson’s Disease [75], synucleopathies [76], and beta-amyloid peptide-induced impairments [74].

The molecular mechanisms that might be associated with the potential use of FTY-720 to induce apoptosis in cancer cells from a variety of neuronal and non-neuronal origins are not fully understood. In relationship to neuronal function, effects limiting the proliferation of neuroblastoma [77], glioblastoma [78], and medulloblastoma cells [79] have been reported. Non-neuronal cancers such as, leukaemia [80], breast [81], melanoma [82], and others, seems also to benefit from such anti-proliferative effects. It is believed that the apoptosis is of mitochondrial origin, involving the dramatic reduction of mitochondrial membrane potential, the release of cytochrome c, and activation of caspases [85], and in consequence activation of the apoptotic cascade. It is also evident that PP2A activity is induced by FTY-720 in many cases [86]. However, we propose here that direct activation of mitochondrial apoptotic signals when the membrane potential is altered after the fusion of organelles is an alternative mechanism to consider, especially in the case of tumours of neuronal and neuroendocrine origins.

Taken together, it is now evident that sphingolipids, their derivatives and related drugs such as FTY-720 could act directly upon a variety of cellular targets that affect membrane fusion processes associated with neurotransmitter release by exocytosis and also that affect intracellular transport and organelle fusion and fission. These newly discovered molecular mechanisms may help to explain the ability of sphingolipids to influence the course of neuronal syndromes and even cancer in neuronal and other tissue models.

In conclusion, studies of the molecular effects of sphingolipids on membrane fusion and related processes increase our understanding of the physiological regulation of neurosecretion and provide a promise to conceive new therapeutic approaches against neurological syndromes [67,68,69,70,71,72,73,74,75,76,90], and cancer [77,78,79,80,81,82,83,91].
